# Pollination and Plant Reproductive Success of Two Ploidy Levels in Red Clover (*Trifolium pratense* L.)

**DOI:** 10.3389/fpls.2021.720069

**Published:** 2021-08-05

**Authors:** Shuxuan Jing, Per Kryger, Bo Markussen, Birte Boelt

**Affiliations:** ^1^Department of Agroecology, Aarhus University, Slagelse, Denmark; ^2^Department of Mathematical Sciences, University of Copenhagen, Copenhagen, Denmark

**Keywords:** plant reproduction, seed production, polyploidy, visitation rate, hand pollination

## Abstract

Plant reproduction in red clover requires cross-fertilization via insect pollination. However, the influences of visitation rate and timing on maximizing ovule utilization are yet to be determined. We aimed to study the influences of visitation rate, flowering stage, and self-incompatibility on reproductive success. We applied hand and honey bee pollination in the study of eight red clover cultivars with two ploidy levels released between 1964 and 2001. In hand pollination, increasing the visitation rates (from 10 to 80 pollinated florets per flower head) increased the seed number per flower head but reduced the seed number per pollinated floret. Different flowering stages (early, middle, and full flowering) did not influence the seed number per pollinated floret significantly. There was a marked difference in reproductive success depending on the ploidy level, with 0.52 seeds per pollinated floret in diploid and 0.16 in tetraploid cultivars. During the cultivar release history, seed number per pollinated floret seemed to decrease in diploid cultivars, whereas it increased in tetraploids. In honey bee pollination, diploid cultivars had more two-seeded florets than tetraploids. Different visitation rates and the stochastic nature of pollen transfer resulted in difficulties when the plant reproductive success between hand and bee pollination was compared. A maximum of 0.27 seeds per pollinated floret were produced in hand pollination compared to the 0.34 in honey bee pollination. In spite of this, hand pollination provided a valuable method for studying the pollination biology and reproduction of red clover. Future studies may employ hand pollination to unravel further aspects of the low reproductive success with the future perspective of improving seed number per pollinated floret in tetraploid red clover.

## Introduction

The influence of pollinator performance on the plant reproductive success is of interest in the research fields of conservation biology, evolutionary ecology, breeding, and seed/fruit production in agriculture (reviewed in Mayer et al., 2011). The maximal plant reproductive success may potentially be realized by pollinators transferring high-quality pollen grains (i.e., conspecific, compatible, and viable) to receptive stigmas (Ne'eman et al., 2010). Pollinator performance can be evaluated via the visitation rate, which is defined as the visit activity of the individual pollinator or simply by the relative abundance of pollinators. However, the visitation patterns are difficult to measure due to the preferences and behavior of the pollinators in relation to varied floral traits and rewards under natural conditions (Ne'eman et al., 2010). The recommendations for crop pollination management are mostly focused on large fields, where the number of honey bee hives located in or near the crop production field can be optimized (Garibaldi et al., 2020). For example, the recommendation for red clover seed production is using four to five honey bee (*Apis mellifera* L.) colonies per hectare (Brødsgaard and Hansen, 2002). Still, there is a lack of direct measurements at the pollination level, such as the flower visitation rate to estimate pollinator performance in order to maximize the reproductive success of different crops (Garibaldi et al., 2020).

One flower may need only a few bee visits for the maximal ovule development, since subsequent visits with pollen transfer will result in additional seeds (saturation) or even lead to damage of the flower styles (Hegland, 2014; Sáez et al., 2014), showing that total coverage of all flowers is rarely the optimal visitation rate for the plant reproductive success. Still, we have a gap of knowledge concerning the ideal pollination regime for most outcrossing plant species (Brosi et al., 2008; Robin et al., 2019).

Pollination at a certain flowering stage may influence reproductive success, as it is related to the temporal patterns of resource allocation. There might be an overlap between the seed development of the first open flowers and the flowering of the subsequent flowers (Wesselingh, 2007). For example, for plant species with indeterminate flowering, early developing fruits can inhibit further fruit maturation and even subsequent flower development, resulting in the first-fruit dominance (Stephenson et al., 1988). To determine the most favorable timing for pollination, it is relevant to investigate plant reproductive success across different flowering stages.

Red clover (*Trifolium pratense* L.) is an important forage legume that is grown in pure stand or mixed with grasses (Boller et al., 2010). The pollination of red clover, as a cross-pollinated species with high self-incompatibility, relies on the insect pollinators, especially long-tongued bumble bee (*Bombus* spp.) species (Holm, 1966; Goulson et al., 2005). The inflorescence of red clover is a flower head containing ~100 florets (Puri and Laidlaw, 1983; Boelt et al., 2015). The unpollinated flower head remains receptive for pollination for up to 10 days (Free, 1993; Boelt et al., 2015). Each floret can potentially produce two seeds since each ovary has two ovules, but usually, only a single seed is developed (Lorenzetti, 1993; Taylor and Quesenberry, 1996). Since red clover is a fodder plant, high forage yield and field persistence have been the main breeding goals. By contrast, the selection for seed yield is mostly neglected during breeding activities; hence, new cultivars may have poorer seed potential compared to older cultivars (Lorenzetti, 1993; Boller et al., 2010; Boelt et al., 2015). A comparison of reproductive success between the red clover cultivars released from different years may reflect this priority of vegetative traits and help to identify important reproductive traits, which have been neglected but should be included in future selection during breeding activities.

Diploid (2n = 2x =14) is the natural ploidy form of red clover, but tetraploid (2n = 4x = 28) red clover has been produced since the 1940s by using different methods (e.g., colchicine, nitrous oxide, and unreduced gametes) to double the chromosome sets with the aim of improving forage yield, disease resistance, and persistence (Taylor and Quesenberry, 1996). However, polyploid genotypes usually have lower seed yield and fertility compared to diploids. For example, tetraploid watermelon (*Citrullus lanatus*) obtained only 11.7% of seed per fruit compared to diploids (Jaskani et al., 2005). Similarly, the seed yield in tetraploid red clover is usually lower compared to diploid red clover, due to the reduced fertility, low pollen quality, meiotic aberrations, and embryo abortions (Taylor and Quesenberry, 1996; Büyükkartal, 2003, 2007; Vleugels et al., 2019a,b). The doubling of chromosome sets may disturb the self-incompatibility, which is controlled by the S-alleles (Taylor and Quesenberry, 1996; Amdahl et al., 2016; Vleugels et al., 2019a). In neotetraploids, reduced plant reproductive success has been linked to larger variations in the self-incompatibility expression compared to diploids (Siopa et al., 2020). However, for high seed-yielding tetraploid red clover genotypes, a high self-pollination rate of 83% on average was found (Vleugels et al., 2019b). Therefore, it is relevant to compare the self-incompatibility between diploid and tetraploid red clover.

The selection for forage yield may result in changes of floral traits (i.e., larger flower heads and deeper florets) and/or in pollinator rewards in polyploidy plants; it may also modify ecological interactions and lead to shifts in pollinator behavior (Rezende et al., 2020). Nowadays, nearly half of the red clover fields grown for seed production in Sweden are tetraploids. Yet, whether the cultivation of tetraploid red clover has impacted the shifts in the bumble bee community is unclear (Bommarco et al., 2012). It is difficult to determine the role of pollination in explaining seed yield differences between diploid and tetraploid red clover due to the complex field conditions (Vanommeslaeghe et al., 2018; Vleugels et al., 2019a). To evaluate the potential influence of polyploidization in plant-pollinator interactions, it is crucial to have knowledge of how the self-incompatibility differs between autopolyploid and natural diploid plants under defined pollination conditions.

Hand pollination is a useful method when studying the reproductive success (Boller et al., 2010), but few hand pollination studies have been conducted in red clover (e.g., Battle, 1949; Vleugels et al., 2019b), most likely due to the complex floral structure and small and numerous florets in red clover. Still, hand pollination at the floret level may provide a baseline to understand the pollination biology and reproductive success of red clover. In a previous hand pollination study, we found that the increased visitation rate may decrease the average seed set per floret in red clover (Jing et al., 2019), but this conclusion needs to be verified with a larger number of red clover cultivars. In order to increase our knowledge for understanding the differences in reproductive success in relation to ploidy levels, we investigated the influences of visitation rate and flowering stage on the reproductive success in red clover. We used hand pollination under laboratory conditions compared with honey bees for open pollination in a confined environment. This study is based on the following specific hypotheses:

(1) Increasing visitation rates (10, 20, 40, and 80 pollinated florets per flower head) may increase the reproductive success.
(2) Pollination at different flowering stages (early, middle, and full flowering) may influence the reproductive success.
(3) Self-incompatibility (self-pollination and interploidy-pollination) differs between diploid and tetraploid red clover cultivars released in different years.

## Materials and Methods

### Study Site and Plant Material

Experiments were performed at Aarhus University in Flakkebjerg, Denmark (55′19′52”N, 11′24′29”E) from 2018 to 2020. To compare diploid and tetraploid red clover cultivars with different breeding histories, we used eight red clover cultivars (Table 1) released in different years (from 1964 to 2001) and with different ploidy levels (diploid and tetraploid). The cultivar information is shown in Table 1. Seeds were germinated on the moist papers in Petri dishes and placed in a germination chamber with a temperature of 20°C (day and night) and 8-h daylight during November of both 2018 and 2019. For each cultivar, 25 small pots (0.5 L) were prepared for the seedling growth (two seedlings per pot) during winter, located in a cold room with a temperature of 4°C. During the spring of 2019 and 2020, 15 plants per cultivar were transplanted into large pots (5 L) and moved to a semi-field protected from rain and wind. Plants were irrigated daily with the drip irrigation system.

**Table 1:** Plant materials information.

**Cultivar name**	**Ploidy level**	**Released year**	**Seed source**	**Origin country**
Krano Pajbjerg	2x	1967	NordGen	Denmark
Rajah	2x	1983	NordGen	Denmark
SW Ares	2x	2001	Lantmännen Lantbruk	Sweden
Suez	2x	2001	DLF	Czech Republic
Hera Pajbjerg	4x	1964	NordGen	Denmark
Tero	4x	1979	NordGen	Denmark
Amos	4x	2000	DLF	Czech Republic
SW Nancy	4x	2001	Lantmännen Lantbruk	Sweden

### Hand Pollination

Hand pollination experiments were conducted during the summers of 2019 (visitation rate and self-incompatibility) and 2020 (flowering stage). We conducted experiments in a climate chamber with a set-up of 14-h daylight (08:00–22:00) and the temperature regime of 20°C (day) and 15°C (night) (Bowley et al., 1987). Hand pollination followed the protocol by Boller et al. (2010). Pollen grains from pollen donor plants were collected freshly by tripping the pollen from the anthers to the Petri dish with a tiny spoon. Keel and wing petals of the floret were removed using a tweezer. A toothpick was then used to collect pollen grains from the Petri dish and deposit them onto the stigma of the floret. New pollen grains were collected on the toothpicks for every 10 florets, and a new toothpick was used for each flower head. We deposited ~40 pollen grains per stigma. Pollen germination test (i.e., percentage of germinated pollen grains out of the total pollen grains) was conducted for each red clover cultivar *in vitro* using culture medium (100 ml culture medium: 25 g sucrose, 2 g agar, and 100 ml distilled water), and pollen grains were incubated overnight (12 h) at room temperature in the dark (Elçi, 1982; Büyükkartal, 2003). Four plants per cultivar were tested for pollen germination. For each plant, pollen grains from three flower heads were tested (on average, 41.2 pollen grains per flower head).

#### Visitation Rate

To study plant reproductive success under different visitation rates, we aimed for five plants per cultivar (however, only four plants in “Rajah” and three plants in “Ares” and “Nancy” were available for this experiment). For each plant, we hand-pollinated 12 flower heads with four treatments (10, 20, 40, and 80 pollinated florets per flower head). Each treatment was applied to three flower heads per plant. For the treatment of 80 florets per flower head, all the available florets were pollinated if the maximum floret number was lower than 80. Pollen donor plants in the visitation rate experiment were different plants (three to four plants) from the same cultivar grown in the climate chamber.

#### Flowering Stage

**Figure 1:**
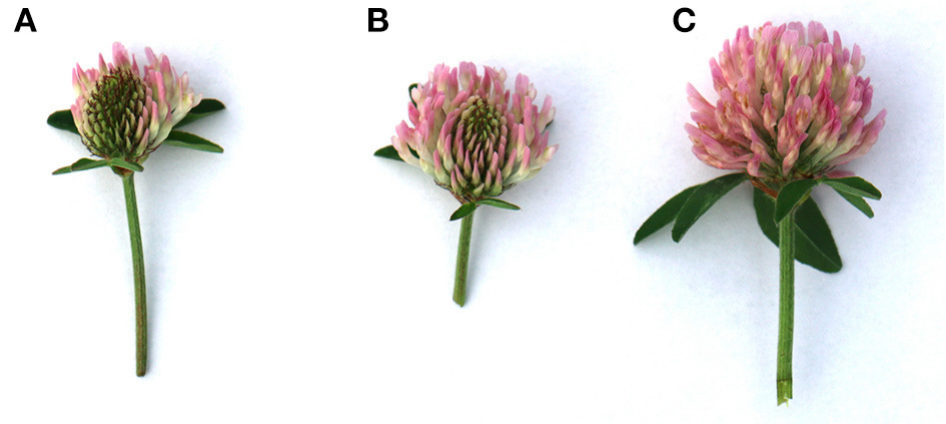
Different flowering stages of red clover flower heads: **(A)** early flowering—when the first 20 florets per flower head were open; **(B)** middle flowering—when nearly half of the florets per flower head were open; and **(C)** full flowering—when all of the florets per flower head were open.

To study plant reproductive success under different flowering stages, we used five plants per cultivar. For each plant, we used nine flower heads with three treatments (early, middle, and full flowering). Each treatment was applied to three flower heads per plant. For each flower head, we hand-pollinated 20 florets. Early, middle, and full flowering stages were defined by the opening of the first 20 florets, nearly half of the florets, and all florets on each flower head, respectively (Figure 1). Pollen donor plants in the flowering stage experiment were different plants (three to five plants) from the same cultivar grown in the climate chamber.

#### Self-Incompatibility

To study self-incompatibility among different red clover cultivars, we used five plants per cultivar. It is important to differentiate between autonomous self-pollination (i.e., in the absence of pollen transfer) and self-pollination (i.e., pollen transferred from the same plant) (Wesselingh, 2007). For each plant, we used nine flower heads with three treatments: control without pollen transfer, self-pollination, and interploidy-pollination. Each treatment was applied to three flower heads. In the control treatment, we removed keel and wing petals of 20 florets per flower head by using a tweezer, carefully avoiding depositing pollen grains on the style. Due to the small floret size and large sample size, any attempts to emasculate the florets were deemed impossible. In self-pollination, we used pollen grains from the same plant. In interploidy-pollination, we pair-grouped red clover cultivars with shared flowering time (by observation) but different ploidy level. We used pollen grains from three to five plants from the other pair-grouped cultivar. Specifically, eight cultivars were pair-grouped into four groups: “Suez” and “Amos,” “Krano Pajbjerg” and “Hera Pajbjerg,” “SW Ares” and “SW Nancy,” and “Rajah” and “Tero.”

### Honey Bee Pollination

We used honey bee pollination as a control for insect pollination. Honey bee pollination experiments were conducted in a confined environment, i.e., a tunnel covered with plastic during summer 2020. One small honey bee colony with around 1,500 honey bee individuals in a mating nuc (Büchler et al., 2013) was located in the tunnel as pollinators. We prepared an adequate number of flower heads by moving 10 plants per cultivar from the semi-field into a pollinator-free climate chamber prior to the honey bee experiment. Afterward, we moved these 10 plants into the tunnel as pollen donor plants when more than 10 flower heads per plant were fully open. Each cultivar was open-pollinated by honey bees for two full days. In total, six red clover cultivars were included in the honey bee pollination. Red clover cultivar “Amos” and “SW Nancy” were not included due to the lack of suitable flower heads after the hand pollination experiments.

### Plant Reproductive Success

Following the hand pollination experiment, the pollinated flower heads were carefully bagged using paper bags that had one side with tracing paper (for light transparency) and the other with cellophane with air-permeable small holes. After the honey bee pollination, we bagged 10 flower heads per plant on five plants per cultivar. All pollinated plants were moved to the semi-field for seed development.

Plant reproductive success was measured after the flower heads had been harvested, air-dried, hand threshed, and cleaned during October of 2019 and 2020. Each floret can produce maximum two seeds per floret (Lorenzetti, 1993; Taylor and Quesenberry, 1996). However, there is a lack of studies on red clover reporting seed number at the floret level (reviewed in Jing et al., 2021). Therefore, in this study, we analyzed all pollinated florets per flower head, and for each floret, we checked whether the floret produced zero, one, or two seeds. Additionally, we recorded the number of florets per flower head from all the harvested flower heads.

### Statistical Analysis

To test how the plant reproductive success was influenced by visitation rate (hypothesis 1), we analyzed seed numbers both at flower head and floret levels. At the flower head level, we fitted generalized linear mixed model (GLMM) to seed number per flower head (Poisson distribution with a log link). At the floret level, we used the cumulative link mixed model (CLMM) fitted with the Laplace approximation to seed number per pollinated floret. Seed number per pollinated floret was the response variable with three ordinally ranked classes (0, 1, and 2) as the possible number of seeds developed per floret can be 0, 1, or 2. For both analyses at flower head and floret levels, we included fixed effects of visitation rate (10, 20, 40, and 80), ploidy (diploid and tetraploid), released year (before 2000 and after 2000), and their interactions. Cultivar (*N* = 8) and flower head ID (*N* = 417) nested within plant ID (*N* = 35) were the random effects.

To test how the stages of flowering influenced the red clover plant reproductive success (hypothesis 2), we fitted CLMM to seed number per pollinated floret. We included fixed effects of flowering stage (early, middle, and full), ploidy (diploid and tetraploid), released year (before 2000 and after 2000), and their interactions. We also included random effects of cultivar (*N* = 8) and flower head ID (*N* = 343) nested within plant ID (*N* = 40). For both CLMMs of visitation rate and flowering stage, we conducted model selection with single term deletions, and we excluded non-significant interactions from the model.

To study the self-incompatibility of red clover (hypothesis 3), we fitted GLMM to the seed number per flower head (Poisson distribution with a log link). Fixed effects were self-incompatibility (self-pollination and interploidy-pollination), ploidy (diploid and tetraploid), released year (before 2000 and after 2000), and their interactions. We did not obtain the data from cultivar “Ares” due to lack of plant material. Random effects were cultivar (*N* = 7) and flower head ID (*N* = 186) nested within plant ID (*N* = 31). Furthermore, we compared the difference between autonomous self-pollination and self-pollination. We fitted GLMM to seed number per flower head (Poisson distribution with a log link). Fixed effects were treatment (control and self-pollination), ploidy (diploid and tetraploid), released year (before 2000 and after 2000), and their interactions. Random effects were cultivar (*N* = 7) and flower head ID (*N* = 186) nested within plant ID (*N* = 31).

In honey bee pollination, we compared the seed number per flower head between one-seeded and two-seeded florets in diploid and tetraploid red clover by fitting GLMM (Poisson distribution with a log link). Fixed effects were floret (one-seeded and two-seeded floret), ploidy (diploid and tetraploid), and their interactions. We did not include the effect of released year in the analysis due to lack of data from cultivars “Amo” and “SW Nancy” (i.e., absence of tetraploid cultivars released after 2000). As we did not know the actual number of florets pollinated by the honey bees, we included the logarithm of number of florets per flower head as an offset variable in the GLMM. This corresponded to the assumption that a constant proportion of all florets were pollinated. Random effects were cultivar (*N* = 6) and flower head ID (*N* = 284) nested within plant ID (*N* = 29). To further compare the seed number per pollinated floret in honey bee pollination with the visitation rate experiment, we fitted CLMM to seed number per pollinated floret to obtain the ordinal responses including fixed and random effects previously described. We fitted GLMM to compare pollen germination in red clover cultivars. Fixed effects were ploidy (diploid and tetraploid), released year (before 2000 and after 2000), and their interactions. Random effects were cultivar (*N* = 8) and flower head ID (*N* = 96) nested within plant ID (*N* = 32).

All statistical analyses and model fittings were performed using the software R version 4.0.3 (R Core Team, 2020). We fitted ordinal regression and Poisson regression by using R packages “ordinal” (Christensen, 2020) and “lme4” (Bates et al., 2015). In model selections, we excluded interactions that were not significant from the analysis. Statistical significance of effects was obtained as type III tests using “drop1()” in R. Estimated marginal means (EMMs) were compared for the significant fixed effects and interaction effects by using *post-hoc* tests in R packages “multcompView” (Graves et al., 2019) and “emmeans” (Lenth, 2020). When comparing more than two EMMs, the *p*-values were adjusted for multiple comparisons, which will be indicated by “adjusted *P*” in the text.

## Results

### Visitation Rate

**Figure 2:**
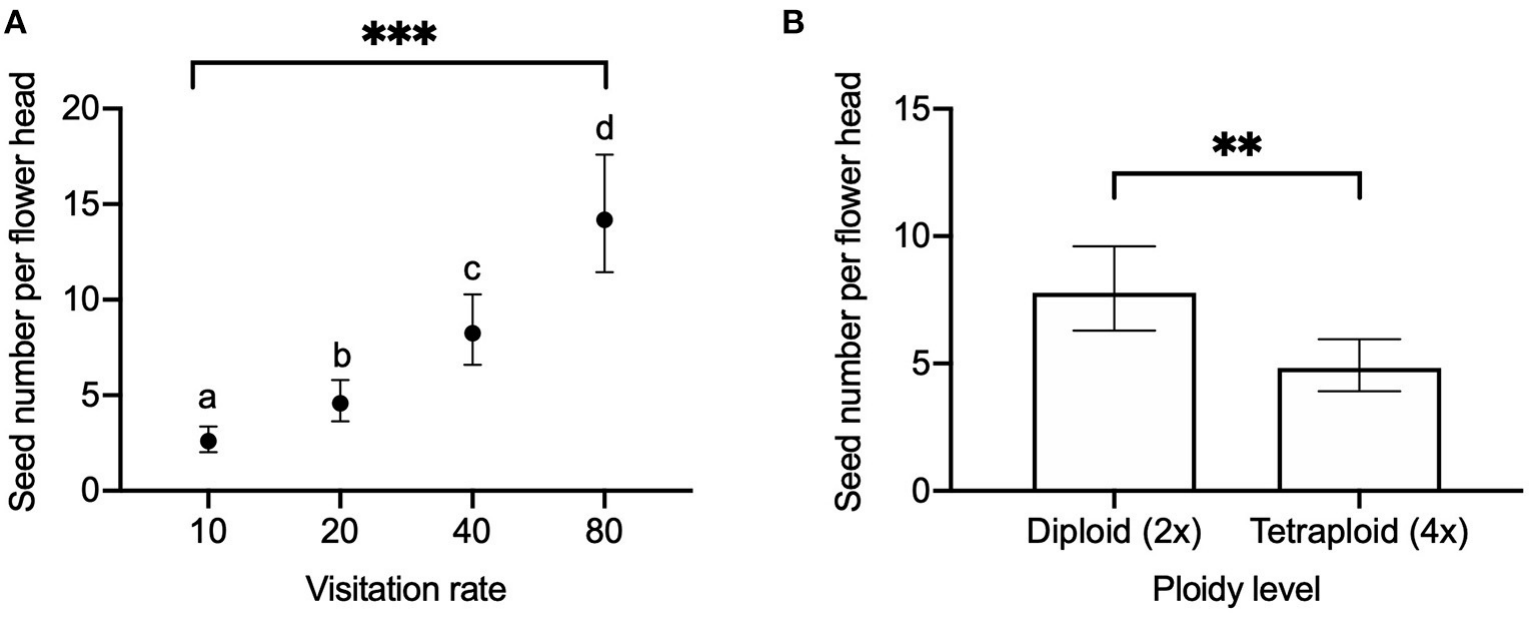
Comparison of seed number per flower head in red clover under **(A)** different visitation rates (10, 20, 40, and 80 pollinated florets per flower head) and **(B)** comparison between diploid and tetraploid in hand pollination. Error bars indicate the 95% confidence interval of the estimated marginal means (EMMs). EMMs sharing a lowercase letter within the same figure are not significantly different at 0.05 significance level. **P value = 0.0090; ***P-value < 0.0001.

**Figure 3:**
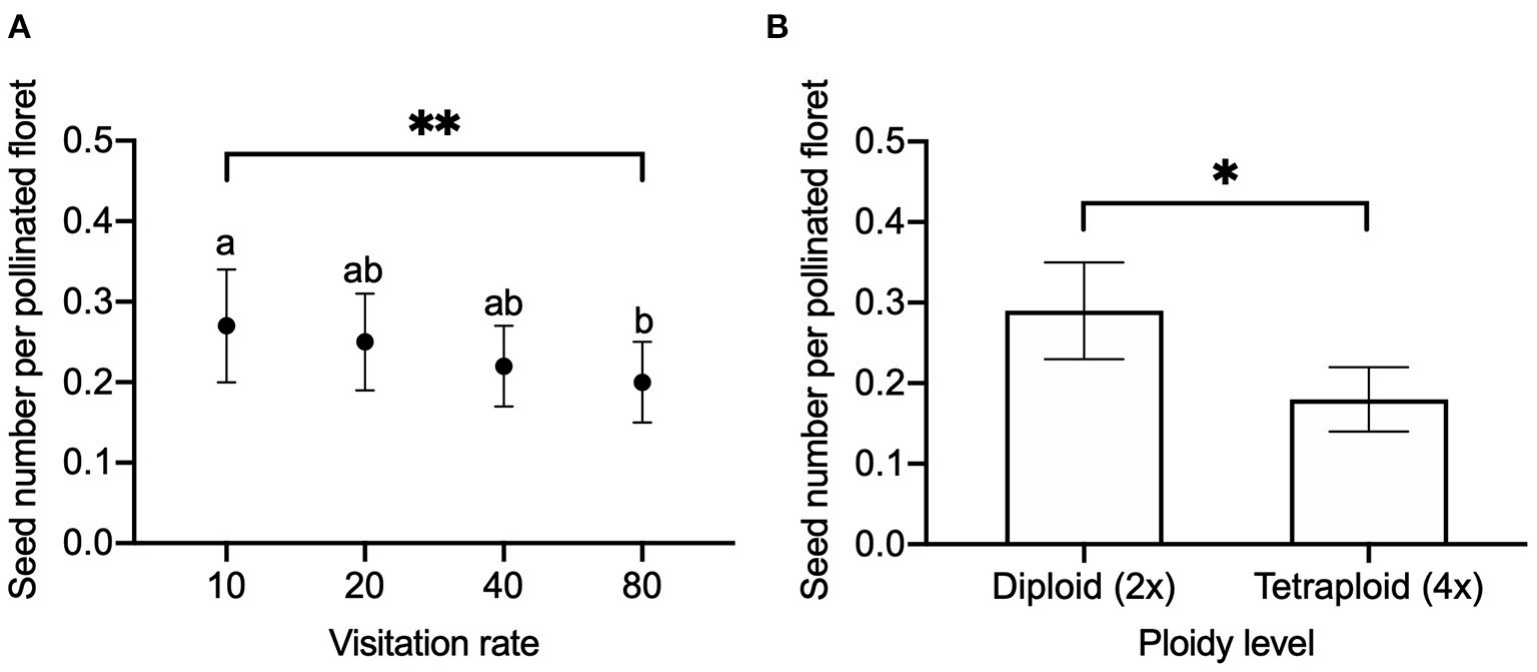
Comparison of seed number produced per pollinated floret in red clover under **(A)** different visitation rates (10, 20, 40, and 80 pollinated florets per flower head) and **(B)** comparison between diploid and tetraploid in hand pollination. Error bars indicate the 95% confidence interval of the estimated marginal means (EMMs). EMMs sharing a lowercase letter within the same figure are not significantly different at 0.05 significance level. *P-value = 0.0170; **P-value = 0.0083.

We found a significant effect of visitation rate and ploidy on the seed number per flower head (Figure 2) and seed number per pollinated floret (Figure 3), while there was no significant effect of released year (Table 2). Seed number per flower head increased with the visitation rate (Figure 2A). Seed number per flower head in diploids [EMMs 7.76 with 95% CI (6.28, 9.59)] was significantly (*P* = 0.009) higher than that of tetraploid red clover [EMMs 4.83 with 95% CI (3.91, 5.95), Figure 2B]. Seed number per pollinated floret at the visitation rate of 80 [EMMs 0.20 with 95% CI (0.15, 0.25)] was significantly (adjusted *P* = 0.0123) lower compared with the pollination of 10 [EMMs 0.27 with 95% CI (0.20, 0.34), Figure 3A]. Seed number per pollinated floret in diploids (EMMs 0.29 with 95% CI [0.23, 0.35]) was significantly (*P* = 0.0043) higher than that of tetraploid red clover [EMMs 0.18 with 95% CI (0.14, 0.22)], Figure 3B].

**Table 2:** The effects of visitation rate, ploidy, and released year on the seed number per flower head [generalized linear mixed model (GLMM)] and seed number per pollinated floret [cumulative link mixed model (CLMM)].

	**Seed number per flower head**			**Seed number per pollinated floret**		
**Term**	**LRT**	**df**	***P-*value**	**LRT**	**df**	***P-*value**
Visitation rate	289.563	3	** <0.0001**	11.760	3	**0.0083**
Ploidy	6.821	1	**0.0090**	5.701	1	**0.0170**
Released year	1.013	1	0.3141	0.817	1	0.3661

*LRT, likelihood ratio test statistic. P-values below 0.05 are shown in bold*.

### Flowering Stage

**Figure 4:**
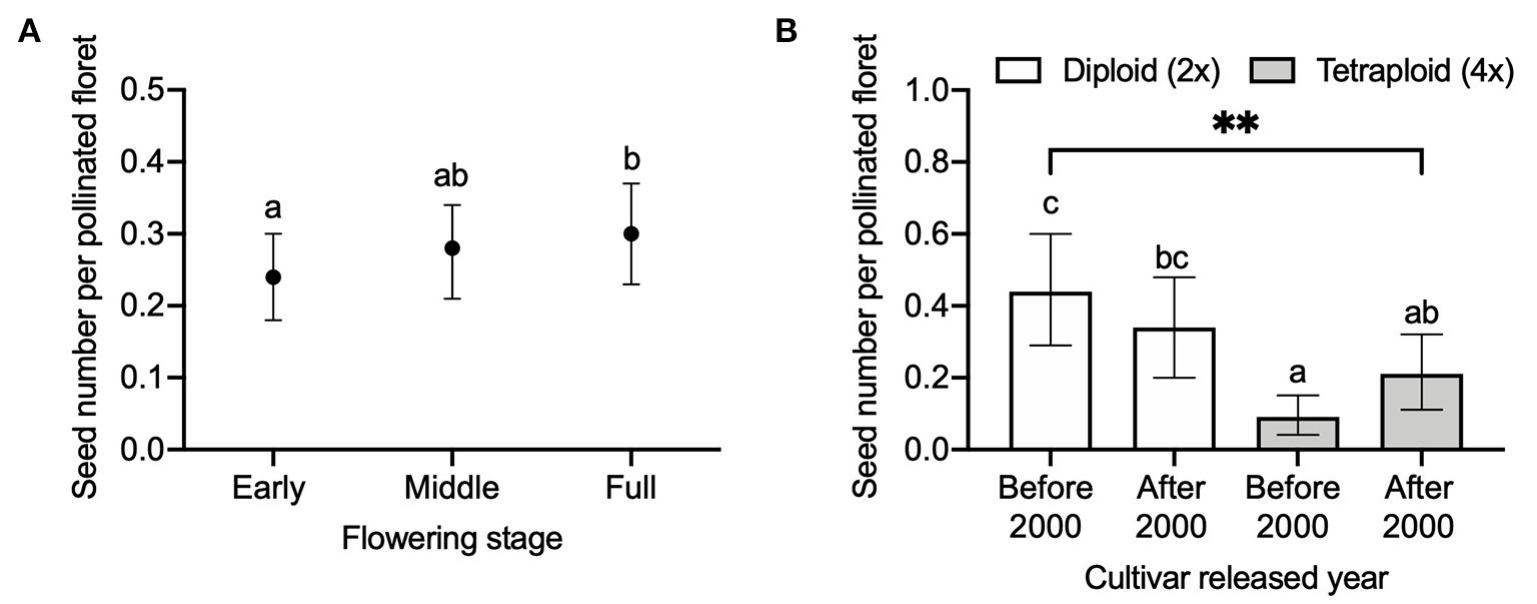
Comparison of seed number per pollinated floret **(A)** among different flowering stages of early, middle, and full and **(B)** between diploid (2×) and tetraploid (4×) red clover cultivars released before 2000 and after 2000. Error bars indicate the 95% confidence interval of the estimated marginal means (EMMs). EMMs sharing a lowercase letter within the same figure are not significantly different at 0.05 significance level. **P-value = 0.0075.

When analyzing the effect of flowering stage on seed number per pollinated floret, we found a significant (*P* = 0.0075) interaction between ploidy and released year on seed number per pollinated floret (Table 3). Comparing between the ploidy levels, seed number per pollinated floret in diploids released before 2000 was significantly higher than tetraploids released before 2000 (adjusted *P* < 0.0001) and after 2000 (adjusted *P* = 0.0101, Figure 4B). Within the same ploidy level, diploids seemed slightly decreased in seed number per pollinated floret with the cultivar released year, i.e., from 0.4 to 0.3, while it increased in tetraploids, i.e., from 0.1 to 0.2.

**Table 3:** Ordinal regression of cumulative link mixed model (CLMM) (LRT, likelihood ratio test statistic) for the effects of flowering stage, ploidy, and released year on the seed number per pollinated floret in red clover.

**Term**	**LRT**	**df**	***P-*value**
Flowering stage	5.6512	2	0.0592
Ploidy × released year	7.1572	1	**0.0075**

*P-values < 0.05 are shown in bold*.

When 20 florets per flower head were hand-pollinated at different flowering stages, we found that the overall effect of flowering stage on seed number per pollinated floret was not significant (*P* = 0.0592, Table 3), but we found that seed number at the early [0.24 with 95% CI (0.19, 0.29)] flowering stage was significantly lower than that of full open [0.30 with 95% CI (0.24, 0.36)] flowering stage (adjusted *P* = 0.0477, Figure 4A).

### Self-Incompatibility

**Figure 5:**
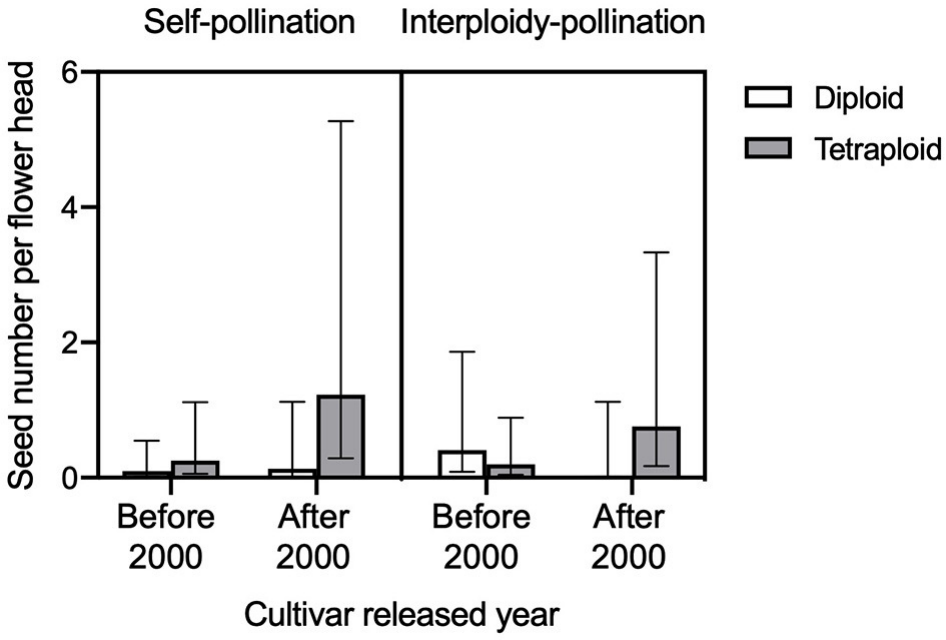
Comparison of seed number per flower head in diploid and tetraploid with different released year (before 2000 and after 2000) under self-pollination (left) and interploidy-pollination (right). Error bars indicate the 95% confidence interval of the estimated marginal means (EMMs).

**Figure 6:**
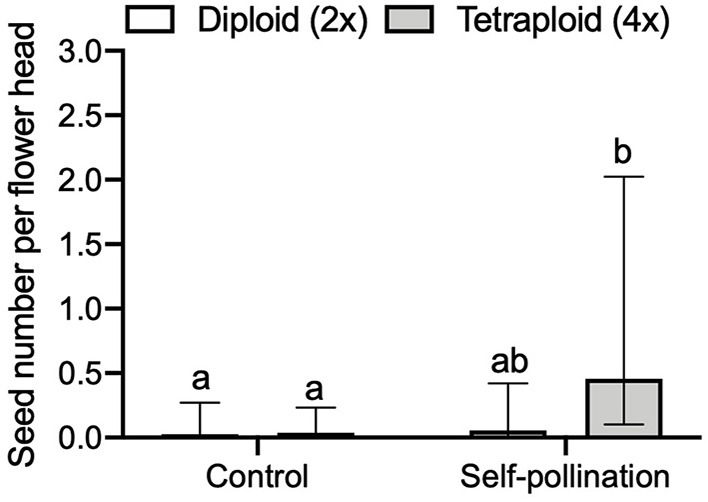
Comparison of seed number per flower head between control (no pollen transfer) and self-pollination in red clover. Error bars indicate the 95% confidence interval of the estimated marginal means (EMMs). EMMs sharing a lowercase letter within the same figure are not significantly different at 0.05 significance level.

We found a significant three-way interaction effect of self-incompatibility, ploidy level, and released year on the seed number per flower head (GLMM, LRT = 15.533, df = 7, *P* = 0.0297, Figure 5). For the comparison of seed number per flower head between control (no pollen transfer) and self-pollination, we found a significant interaction effect of treatment and ploidy (GLMM, LRT = 4.195, df = 1, *P* = 0.041). In tetraploid red clover, the seed number per flower head in self-pollination was significantly (adjusted *P* < 0.0001) higher compared with the control (Figure 6). No significant effect of released year was found in the current comparison (GLMM, LRT = 1.319, df = 1, *P* = 0.251).

### Pollen Germination

We found that there were no significant effects of ploidy (GLMM, LRT = 2.245, df = 1, *P* = 0.134) and released year (GLMM, LRT = 0.00003, df = 1, *P* = 0.9958) on the pollen germination. The average pollen germination in diploid red clover cultivars was 0.634 with 95% CI (0.496, 0.772) and 0.455 with 95% CI (0.304, 0.615) in tetraploid cultivars.

### Honey Bee Pollination

**Figure 7:**
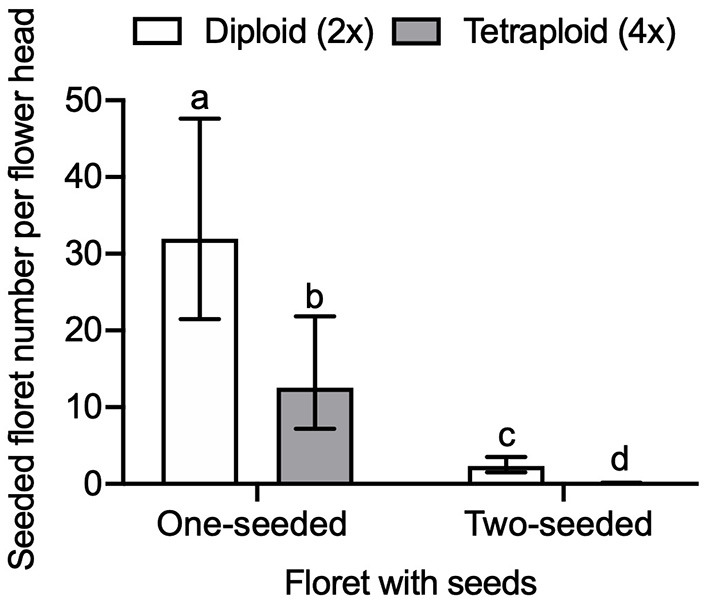
Comparison of seeded floret number per flower head between diploid (2×) and tetraploid (4×) red clover cultivars under honey bee pollination. Error bars indicate the 95% confidence interval of the estimated marginal means (EMMs). EMMs sharing a lowercase letter within the same figure are not significantly different at 0.05 significance level.

We found a significant interaction effect of seeded floret and ploidy on the seed number per flower head (GLMM, LRT = 136.17, df = 1, *P* < 0.0001). The number of one-seeded and two-seeded florets differed between diploid and tetraploid red clover (Figure 7). The number of seeded florets in diploids was significantly higher than tetraploids in both one-seeded (adjusted *P* = 0.0036) and two-seeded florets (adjusted *P* < 0.0001). The number of two-seeded florets in tetraploid red clover was close to 0 [0.061 with 95% CI (0.02, 0.18)]. The number of two-seeded florets of the total seeded florets in diploid red clover was 7.27% with 95% CI (7.18, 7.37). The overall seed number per pollinated floret was 0.34 with 95% CI (0.24, 0.44). In diploid red clover, the average seed number per pollinated floret was 0.52 with 95% CI (0.36, 0.69), and 0.16 with 95% CI (0.04, 0.27) in tetraploid.

## Discussion

### Visitation Rate and Flowering Stage

At the flower head level, we found that reproductive success was increased with the visitation rate (Figure 2A), supporting our first hypothesis. However, the floret fertility was decreased with the increasing visitation rate (Figure 3A). For example, we obtained the highest seed set (0.27 seeds per pollinated floret) at the visitation rate of 10 florets per flower head at the full flowering period. However, at the highest visitation rate of 80 florets per flower head, we only obtained 0.20 seeds per pollinated floret. Comparing hand pollination with honey bee pollination, the seed number per pollinated floret in hand pollination was much lower compared to the 0.34 seeds per pollinated floret in honey bee pollination that were used as a control representing insect pollination. Our results showed that the reproductive success in hand pollination was lower than honey bee pollination in this study.

**Figure 8:**
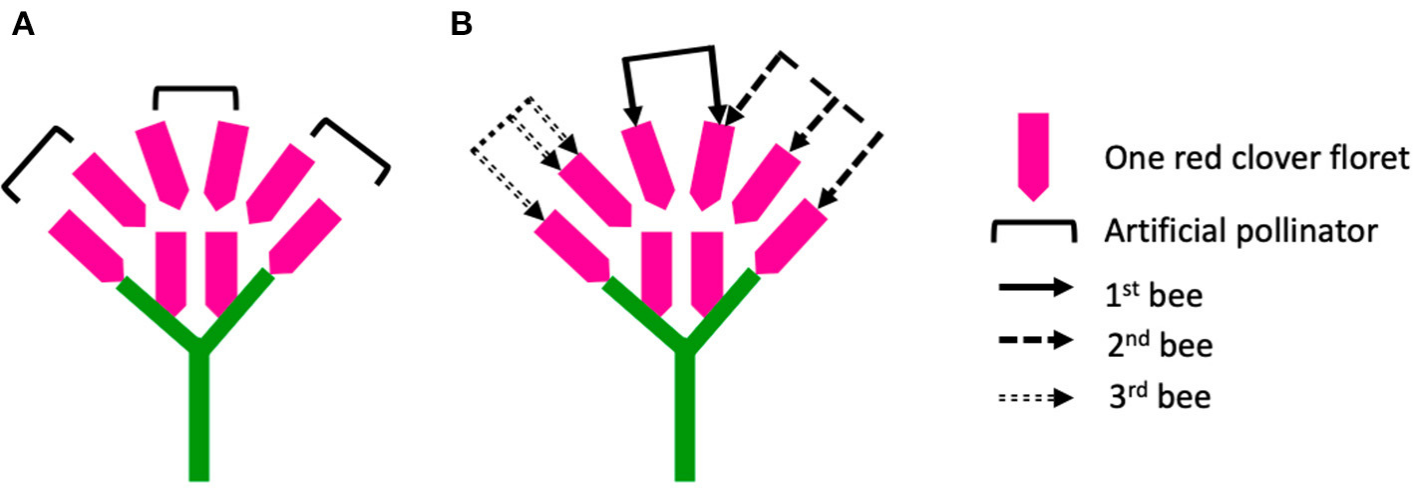
Illustration of a simplified red clover flower head containing eight florets per flower head. Six of the florets were pollinated, but under two different pollination approaches: **(A)** hand pollination, where each artificial pollinator had a visitation rate of two without re-visiting the florets, and **(B)** open pollination (three bees visiting the flower head subsequently), where the first bee had a visitation rate of two by visiting two different florets, the second bee had a visitation rate of three but revisited one floret that had been visited by the first bee, the third bee had a visitation rate of three but revisited one floret that had been visited by itself.

During hand pollination, we replaced the toothpick loaded with fresh pollen grains for every 20 florets. Therefore, we estimated that at the visitation rate of 80 florets per flower head, each flower head had four artificial pollinators (toothpicks) visiting in sequence, without revisiting any previously tripped florets. In this study, we tried to illustrate a scenario to explain the different results from hand pollination vs. honey bee pollination approaches (Figure 8).

We assumed that one red clover flower head contained a total of eight florets per flower head but treated with different pollination approaches (Figure 8). In Figure 8A, the flower head was hand- pollinated at the visitation rate of six florets per flower head by using three toothpicks loaded with fresh pollen grains continuously. In Figure 8B, the flower head also got the visitation rate of six florets per flower head, as the six florets were visited by three bees subsequently. However, the behavior of the bees varied. The first bee visited two different florets per flower head, the second bee visited three florets per flower head but included one floret that had been visited by the first bee, and the third bee visited two different florets per flower head but revisited one floret that itself had already visited. Several factors can also make the scenario even more complicated, namely, the three bees may represent different pollinator species, carry pollen grains differing in quality and quantity, and both add and remove pollen grains on the stigma, thus making the pollen transfer more stochastic compared to the hand pollination. If we simply count the number of visits by different bees, there is a gap between the counted visitation rate of bees (eight visits for three bees) and the actual visitation rate (six florets pollinated per flower head). Therefore, the stochastic pollen transfer by multiple bee visits may explain why the reproductive success in hand pollination was lower than the bee pollination in this study.

The floral structure of red clover, which consists of many florets per flower head, increases the difficulties of correlating the visitation rate of bees with the plant reproductive success and further increases the challenge of estimating the number of pollinators needed for maximal plant reproductive success (Fijen and Kleijn, 2017). Plowright and Hartling (1981) suggested that the correlation of seed set and pollinator behavior cannot be determined by simply estimating the frequencies of pollinator movements between the red clover plants. Using the plant species white clover (*Trifolium repens* L.) with a floral structure similar to red clover, Goodwin et al. (2011) calculated the theoretical potential based on the measured floral traits (e.g., floret number per flower head), observed pollination success (e.g., visitation rate), and found that the actual seed set was only half of the calculated theoretical potential. In addition, they suggested that the contribution of the subsequent bees to plant reproductive success may be lower compared to the first bee (Goodwin et al., 2011).

Furthermore, the floral rewards of nectar and pollen may diminish once the first floret is pollinated, which may reduce the floral attractiveness for subsequent bees and consequently influence the pollination behavior of the bees. For example, the development of embryos in red clover from the spherical to the heart-shaped stage requires 3–5 days after cross-pollination (Armstrong, 1968; Taylor and Quesenberry, 1996). The floral traits may change during the period between the first pollination event and the embryo development. This may explain why we obtained higher seed number per pollinated floret at the visitation rate of 10 compared to 80 (Figure 3). The expectation from the first-fruit dominance hypothesis is that the developing seeds of the first flowers further inhibit either the seed set or the attractiveness of the flowers opening later (Stephenson et al., 1988). The first pollinated florets may restrict the later pollinated florets, and first florets that are pollinated and developing seeds had an advantage (Ladio and Aizen, 1999; Wesselingh, 2007). Pollination at the early flowering stage (with florets only partially open) may also result in a lowered floret number per flower head. Furthermore, the maternal investment of the plant may adjust the reproductive cost for further developing florets due to a limiting resource (Lloyd, 1980). This hypothesis still remains untested in red clover, because in Figure 8, we have only considered the pollination process when the flower heads were fully opened.

Bees may continuously visit florets during the flowering stages under field conditions. In other words, flower heads may already get visited when florets are only partially open. Therefore, the influence of flowering stage on the seed number was explored by hand pollination. We found that the seed number per pollinated floret was higher at the full flowering stage compared with the early flowering stage (Figure 4A). A temporal variation in resource availability may explain the lower seed number per pollinated floret at the early stage compared with the later flowering stage; however, by applying hand pollination, pollen can be excluded as a lacking resource. Lloyd (1980) suggested that plants may adjust their maternal investment during three temporal stages: flower determination, ovary development, and fruit maturation. In the flowering stage experiment, we hand-pollinated flower heads at different flowering stages on the same plant. Here, with pollination during the early flowering time, the overlap between continued flower development and the seed development stage may potentially influence the results of reproductive success among different treatments. While we hand-pollinated 20 florets per flower head at each flowering stage, we did not attempt to explore whether the reproductive success differed according to the spatial arrangement of the red clover. For example, basal florets that developed earlier are potentially stronger resource sinks for developing seeds compared to later developing florets (Vallius, 2000; Wesselingh, 2007). Furthermore, the variations in pollen deposition and resource availability may differ among the individual flowers (Wesselingh, 2007). Jakobsen and Martens (1994) also found that delayed pollination after the anthesis decreased the seed set in white clover. In our study, we only investigated the flowering stage from early (first 20 florets open) to full (all the florets open) flowering stage and avoided any influence from delayed pollination and senescence.

The previous studies indicate that pollinating only a fraction of the flowers may result in misleading plant reproductive success due to the resource allocation (Zimmerman and Pyke, 1988; Knight et al., 2006; Wesselingh, 2007), since the full potential seed set may never be realized. We found that the seed number per pollinated floret was higher when pollinating only a fraction of flowers (visitation rate of 10) compared to the higher visitation rate of 80 (Figure 3). Even though we succeeded in pollinating at the maximal visitation rate per flower head, the results of reproductive success from the hand pollination may still be misleading due to the stochastic pollen transfer by multiple bee visitors (Figure 8). Applying hand pollination to all florets did facilitate reproductive success in red clover well below the maximal ovule development, indicating that pollination is not the only limiting factor. Additional qualified and quantified data on the individual bee behavior are required.

### Ploidy, Cultivar Release Year, and Self-Incompatibility

We found that, in cultivars released before 2000, the seed number per pollinated floret in diploids was significantly (*P* < 0.0001) higher than that of tetraploids (Figure 4B) in the flowering stage experiment, while in cultivars released after 2000, the difference was not significant. These data indicate the achievements in breeding new cultivars with higher seed yield during the recent decades, with seed yield increasing in tetraploids, in particular. Red clover has a low capacity for autonomous self-pollination, while tripping the florets on the flower head may result in producing self-seeds (Williams, 1931; Denward, 1963; Taylor and Quesenberry, 1996). Higher seed number per flower head in self-pollination compared to the control showed that tetraploid red clover had high self-pollination (Figure 6). We also found both diploids and tetraploids were poorly pollinated by pollen with the opposite chromosome set as no significant effect of ploidy on seed numbers was detected (*P* = 0.4128).

High seed-yielding red clover plants have both ovules developing to mature embryo sacs and two mature seeds (Taylor and Quesenberry, 1996). In the current honey bee experiment, the proportion of two-seeded florets out of the total seeded florets in diploid red clover was 7.27%, which is in agreement with the proportion of two-seeded florets in a range of 6–11% found in the previous studies (Dijkstra, 1969; Richards, 2016). However, the number of two-seeded florets in tetraploid red clover was close to 0 [EMMs 0.06 with 95% CI (0.02, 0.18), Figure 7].

Tetraploid red clover have higher forage production, but plant reproductive success is lower compared to diploids, the reasons for which have mostly been attributed to fertility problems such as meiotic aberrations or self-fertility and subsequent inbreeding depression (Vleugels et al., 2019a,b). We found that both the number of one-seeded florets and two-seeded florets in the tetraploids were lower compared to the diploid red clover (Figure 7). We also found that pollen germination rate in tetraploids was lower compared to diploids and that the self-pollination rate in tetraploids was higher than diploids, but both not statistically significant. Vleugels et al. (2019b) investigated the embryo development between diploid and tetraploid red clover populations and found a high degree of embryo abortion between 7 and 14 days after hand pollination in tetraploids, but significant ploidy effect was not found on the number of developing, underdeveloped, or shriveled embryos. Tetraploids had higher abnormalities in meiotic division in male meiosis when compared to diploid red clover, which can potentially result in low pollen quality and embryo abortion (Vleugels et al., 2019b). In contrast to the reproductive success from cross-pollination, we found higher self-pollination and interploidy-pollination in tetraploids compared to diploids, especially in cultivars released after 2000 (Figure 5). Barringer (2007) also found that polyploids were more self-fertile compared to diploids based on the analysis of 235 flowering plant species. Our results support the hypothesis that there might be a leaky self-incompatibility in neotetraploids caused by polyploidization (Siopa et al., 2020). Furthermore, a recent study found that intensive selection for breeding high seed-yielding tetraploid red clover may increase self-pollination (Vleugels et al., 2019b). Thus, the differences in self-pollination between diploids and tetraploids may lead to different breeding strategies in relation to floral traits. For example, increasing the floret number per flower head or flower head per plant may increase the possibility for self-pollination and, thus, oppositely influence the reproductive success of diploids and tetraploids.

It is interesting to note that, compared to cultivars released before 2000, the seed number per pollinated floret in cultivars released after 2000 had decreased in diploids but increased in tetraploids (Figure 4B). It suggests that, with time, the most seed-productive tetraploid cultivars have been promoted; however, it is difficult to understand why the seed number per pollinated floret decreased in diploids. With only eight cultivars included, our results provide preliminary evidence that the fertility of tetraploid red clover has improved with the released year. Further studies are required to elucidate a potential inbreeding depression and the effect of cultivar released years with more genotypes. Studying the meiotic process is important for understanding the polyploidization process of young polyploidy crop species such as oilseed rape (*Brassica napus* L.) (Mason and Batley, 2015; Mason and Snowdon, 2016). With <100 years of using tetraploid red clover in agriculture, there are large potentials for improving our understanding in the seed yield of tetraploids.

### Other Influential Factors in Hand Pollination

The influential factors in hand pollination are worth further discussion, as there are only a few hand pollination studies in red clover available previously (reviewed in Jing et al., 2021). Compared to bee pollination, hand pollination may result in lower or higher reproductive success in different plant species (reviewed in Young and Young, 1992). As we observed lower reproductive success in hand pollination compared to honey bee pollination in the current study, here, we will discuss the reasonings from Young and Young (1992): pollen quantity (crowding or insufficient), pollen quality (low-diversity or inviable), stigma (damaged or low receptivity), and bagging effect.

A preliminary study on pollen supplementation showed that the increase of pollen grains (increased from ~40 to 80 pollen grains deposited on 10 florets per flower head) increased the seed set, especially for the red clover cultivars with high pollen viability (Jing et al., 2019). In this study, we deposited ~40 pollen grains on each stigma. However, we cannot quantify the number of pollen grains deposited on each stigma for the floret due to the large number of florets being hand-pollinated in this study (~3,000 florets per cultivar), making it impossible to emasculate each floret. As shown in Figure 8, the stochastic pollen transfer under the bee pollination may also cause either a crowding of pollen in certain florets or insufficient quantity of pollen grains in others. The pollen grains used in this study were collected from flower heads from three to five plants and were used fresh (within 30 min). The availability of red clover for honey bee pollination was also at least 100 flower heads from 10 plants in each cultivar. Therefore, more pollen donor plants may result in higher pollen quality in honey bee pollination compared to hand pollination. Although not statistically significant, pollen germination in diploid red clover cultivars was, on average, 64%, which is higher than that of tetraploid red clover (46%). There might not be sufficient viable pollen grains deposited on the stigmas; however, if the pollen germination accurately represents the pollen germination on the stigma, the amount of viable pollen deposited in this study should be easily sufficient for two ovules per ovary. Nevertheless, how accurately the measurements of pollen viability and pollen germination can describe and quantify plant reproductive success is not clear. A recent study using apple (*Malus domestica* Borkh) found that visitation from multiple pollinators resulted in the growth of pollen tubes for embryo and seed development more frequently compared to the visitation from a single pollinator, indicating that pollen tube growth from multiple pollinators can be an accurate way to quantify the pollination and reproductive success (Stavert et al., 2020). Our discussion on the influence of stochastic pollen transfer (Figure 8) may also help to explain why pollination success from multiple pollinators may more accurately estimate plant reproductive success. Free (1965) found that seed sets in red clover did not decrease with the increased visitation rate (number of florets per flower head) of the individual bees, which was in contrast to the expectation that the amount of compatible pollen grains might be diluted by the pollen grains coming from the same flower head. This was explained by the large amount of compatible pollen grains from previous plants, preventing a dilution. In this study, we replaced toothpicks loaded with fresh compatible pollen grains to minimize the dilution effect. Florets were pollinated when fully open, so we assumed that the stigmas were receptive. There was no bagging effect since the plants were moved to a pollinator-free climate chamber prior to the flowering. Still, there might be an effect of using a climate chamber, e.g., different light intensity and daylength during the pollination process, which was also a more constant condition compared to the tunnel in honey bee pollination and the natural condition. The growing conditions for the seed development and maturation were the same in the semi-field. The most relevant limitations in this study might be the limited diversity of pollen grains and insufficient viable pollen grains. In white clover, Cowan et al. (2000) found that incompatible pollen grains would not restrict or compete with compatible pollen grains to develop embryos and seeds. Due to the relatively higher self-pollination rate in tetraploid red clover, the competition between incompatible and compatible pollen grains is yet to be studied.

## Conclusions

By the use of hand pollination studies, we found that an increasing visitation rate of up to 80 florets per flower head may increase the seed number per flower head but decrease the seed number per pollinated floret. Pollination at different flowering stages also had little influence on the plant reproductive success. Diploid red clover showed a higher reproductive success compared to tetraploids by producing more than three times as many seeds per pollinated floret. Tetraploid red clover showed a higher self-pollination, as well. The differences in seed number per pollinated floret were larger between diploids and tetraploids in cultivars released before 2000, with seed numbers slightly increasing in tetraploid cultivars released after 2000. Furthermore, we used honey bee pollination in a confined environment as a control for insect pollination. Comparing hand pollination with honey bee pollination showed a 20% decrease in seed number per pollinated floret from hand pollination, which emphasized the importance of high pollen diversity from multiple pollinator individuals. Future studies may focus on how the breeding activities influence the general reproductive success in diploid and tetraploid red clover and how seed number per pollinated floret in tetraploids may be improved.

## Data Availability Statement

The dataset supporting the conclusions of this article can be found in the Dryad Digital Repository https://doi.org/10.5061/dryad.2bvq83bqk (Jing et al., 2021).

## Author Contributions

SJ: conceptualization, data curation, methodology, investigation, formal analysis, visualization, and writing—original draft. PK: resources, methodology, supervision, visualization, and writing—review and editing. BM: formal analysis, visualization, and writing—review and editing. BB: funding acquisition, conceptualization, resources, methodology, supervision, visualization, writing—review and editing, and project administration. All authors contributed to the article and approved the submitted version.

## Conflict of Interest

The authors declare that the research was conducted in the absence of any commercial or financial relationships that could be construed as a potential conflict of interest.

## Publisher's Note

All claims expressed in this article are solely those of the authors and do not necessarily represent those of their affiliated organizations, or those of the publisher, the editors and the reviewers. Any product that may be evaluated in this article, or claim that may be made by its manufacturer, is not guaranteed or endorsed by the publisher.
